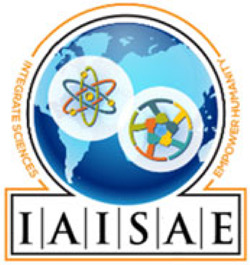# Preface to ‘Thermodynamics 2.0: Bridging the natural and social sciences’

**DOI:** 10.1098/rsta.2022.0274

**Published:** 2023-08-07

**Authors:** Shannon Campbell, Jon McGowan

**Affiliations:** ^1^ Appalachian State University, Boone, NC 28608, USA; ^2^ University of Massachusetts Amherst, MC 28607, USA

## Foreword

On 18–22 July 2022, Appalachian State University hosted the Thermodynamics 2.0 | 2022 Conference in Boone, North Carolina. As Dean of the College of Fine and Applied Arts, it was my pleasure to welcome global thought and practice leaders in the physical, natural and social sciences as well as the humanities to collaborate and engage in transdisciplinary knowledge production and discovery. The purposeful use of the term transdisciplinary rather than interdisciplinary is intentional and accurate. The term interdisciplinary implies a linking and connection of disciplines. The term transdisciplinary suggests a true melding and convergence of disciplines that creates entirely new ways of knowing. Special thanks go to The International Association for the Integration of Science and Engineering (IAISAE) for sponsoring the biennial conference, which created a safe haven for participants to question convention, push the envelope and tackle big issues.

The conference, where partnerships flourished and dots were connected, represented the future of scientific revolution in the natural and social sciences. It also represented the future of academic and appliedapproaches to successfully addressing global challenges and threats to sustainability. It takes courage to *address* universal questions, especially to do so in ways that defy convention; yet that is precisely what readers will find on full display throughout this issue.

The breadth of information shared at the conference mirrors the content of this special issue where topics like quantum systems and foundations, stable evolution, thermodynamics and more are interrogated and investigated. This issue highlights the work of scholars and practitioners deeply committed to addressing the critical issues and challenges facing humanity in the twenty-first century via transdisciplinary approaches. It decodes complexity by highlighting the reliability and connection of natural and physical science, to the vibrancy of social and behavioural science. This transformational issue is vital to the way we understand and connect to the world in which we live and to each other.

I am sure you will find the issue enlightening and thought provoking. The articles leave readers with the lingering question of why not…rather than why? Once we collectively imagine an ideal state and begin to ask the question why not, we begin to successfully change the world. This issue illustrates how we do that together in an environment where collaboration is valued over competition.

Shannon B. Campbell, Ph.D.

Dean

Appalachian State University | College of Fine and Applied Arts 
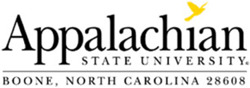


## Preface

Thermodynamics 2.0 is a platform where the natural sciences meet the social sciences. Appalachian State University should be thanked for hosting the Thermodynamics 2.0 conference in the attractive city of Boone, North Carolina. In the summer of 2022, Mike McKenzie (Vice Provost of Academic Program Development and Strategic Initiatives) graciously inaugurated the conference and welcomed presenters and participants from over 26 countries around the world who participated in-person and online. About 80 scholars presented their contributions aimed towards bridging the natural and social sciences. A select number of articles from this 2022 conference are featured in this theme issue by the Royal Society. These articles represent the latest developments in the advancement and applications of the subject of Thermodynamics. It is hoped that they will start many important discussions and interesting debates on this subject that can be applied to solving existing and future problems of the world.

Organizing a conference of this scope and reach is a challenging task in itself. Obtaining and reviewing articles ready for publishing is a separate project. Scholars in natural and social science speak different languages and employ research methodology specific to the academic discipline. Furthermore, bringing a diverse pool of researchers under one roof is a formidable challenge. The organizers met this challenge as reflected by the wide spectrum of participants. This would not have been possible without the careful planning of organizers Ram Poudel, Roshani Silwal and Carey Driskell (Manager, Conference & Event Services at Appalachian State). Brian Raichle and Ok-Youn Yu helped bring the 2022 conference to Appalachian State University. Also, thanks are due to Christine Hendren (Research Institute for Environment, Energy, and Economics (RIEEE)) and Jamie Russell (Appalachian Energy Center) for their encouragement. Georgi Georgiev (Assumption University) organized an inaugural Thermodynamics 2.0 in 2020 at Worcester, Massachusetts. Thus, it should be noted that this international conference has been made possible via a lasting legacy and the support of many others who can only be thanked in collective.

There is no one academic discipline that can tackle the enormous complexity of global challenges faced in the twenty-first century. The contemporary issues range from ecological to economical to environmental and beyond. We need to connect disciplines and combine our strengths. This is a way we can engage in activities beyond the reach and scope of each of us. Connection helps achieve our promise and potential. We get to learn from each other's viewpoints and vocabulary different from ours. A first step along this line could be establishing a common language and building an environment of mutual trust between the people involved. It is hoped that Thermodynamics 2.0 | 2022 at Appalachian State University was successful in this measure.

In summary, the Royal Society editorial team should be thanked for publishing a two-part theme issue on this emerging area of integrated research. The issue bridges traditional science disciplines by bringing together a broad range of contributions. There is no doubt that this landmark resource will be an inspiration to many transdisciplinary scholars and create a lasting resource for academics for many years to come.

Jon McGowan

Board of Directors | IAISAE

Professor | University of Massachusetts Amherst